# Effects of hsa_circ_0074854 on colorectal cancer progression, construction of a circRNA–miRNA–mRNA network, and analysis of immune infiltration

**DOI:** 10.1007/s00432-023-05315-8

**Published:** 2023-08-29

**Authors:** Guida Fang, Dalai Xu, Tao Zhang, Lei Qiu, Xuzhu Gao, Gang Wang, Yongchang Miao

**Affiliations:** 1 Department of Gastrointestinal Surgery, Clinical College of Lianyungang Second People’s Hospital, Bengbu Medical College, Lianyungang, 222002 Jiangsu China; 2 Department of Gastrointestinal Surgery, The Second People’s Hospital of Lianyungang City, Kangda College of Nanjing Medical University, Lianyungang, 222002 Jiangsu China; 3 Institute of Clinical Oncology, The Second People’s Hospital of Lianyungang City (Cancer Hospital of Lianyungang), Lianyungang, 222002 Jiangsu China

**Keywords:** Colorectal cancer, CircRNA, CeRNA network, Immunoinfiltration, Diagnostic and therapeutic biomarker

## Abstract

**Purpose:**

Circular RNAs have been demonstrated to be closely associated with the onset and metastasis of colorectal cancer. However, the roles and clinical diagnostic value of most circRNAs in colorectal cancer remain unclear.

**Methods:**

We detected the differential expression of circRNAs in CRC tissues and cells and investigated their relationship in conjunction with clinical pathological features. Additionally, we performed cellular functional experiments in CRC cell lines to explore the functions of circRNAs. To further validate the potential ceRNA network, qPCR was performed to assess the expression of miRNA and mRNA in CRC cells after differential expression of circRNAs knockdown. Furthermore, database analysis was utilized to explore the relationship between the predicted mRNAs and immune infiltration in CRC.

**Results:**

Our research findings indicate a positive correlation between hsa_circ_0074854 expression and advanced clinical pathological features, as well as an unfavorable prognosis. Knockdown of hsa_circ_0074854 was observed to inhibit proliferation and migration capabilities of colorectal cancer cells, affecting the cell cycle progression, and simultaneously promoting apoptosis. A competing endogenous RNA mechanism may exist among circRNAs, miRNAs, and mRNAs. Furthermore, the expression of target genes displayed correlations with the abundance of certain immune cells.

**Conclusion:**

We propose a novel ceRNA network and evaluate the interplay between target genes and immune cells, providing novel insights for the diagnosis and targeted therapy of CRC.

## Introduction

Colorectal cancer (CRC) is one of the most common malignant tumors in the digestive system and ranks as the second leading cause of cancer-related deaths worldwide (Morgan et al. 2023). With the sustained development of developing countries, the global number of CRC patients is expected to reach 25 million new cases by 2035 (Ait Ouakrim et al. 2015). Incidence and mortality rates of CRC have been progressively increasing in recent years. Emerging evidence suggests a gradual rise in CRC incidence among younger individuals as well (Fang et al. 2023). The majority of patients are diagnosed at an advanced clinical stage, and current primary treatment for CRC remains focused on surgical resection, often accompanied by adjuvant radiotherapy and chemotherapy (Fiala et al. 2019). Despite significant progress in screening, diagnosis, and treatment strategies, the 2-year survival rate for CRC patients remains low (Siegel et al. 2016). The underlying molecular mechanisms of CRC are not yet fully understood (Zhang et al. 2021). In light of this, to extend the survival time of CRC patients, there is a pressing need to actively explore the intrinsic mechanisms of CRC development at the molecular level, elucidate its biological functions, and discover novel molecules relevant to CRC. These discoveries could enable CRC screening, early diagnosis, prognosis, and ultimately facilitate early intervention and treatment to prevent CRC metastasis and improve patient survival rates.

Circular RNA (circRNA) is a type of RNA molecule formed by the covalent binding of the 3′ and 5′ ends to create a closed circular structure (Wang et al. 2021a, b, c). Dysregulation of circRNA expression has been observed in many tumors, making these circRNA expression patterns potentially valuable for diagnostic purposes or as potential therapeutic targets. Functionally, circRNAs can act as competing endogenous RNAs (ceRNAs) that bind and sequester specific microRNAs (miRNAs), thereby preventing their interaction with target mRNA molecules (Pamudurti et al. 2022). While the biological functions of most circRNAs are still not fully understood, an increasing body of evidence supports their critical roles in various disease processes, including neurodegenerative disorders (Dube et al. 2019), metabolic diseases (Xu et al. 2015), and several types of cancer (Qian et al. 2018). For instance, Circ-ASS1 is downregulated in breast cancer cells MDA-MB-231 and can inhibit cell invasion and migration by targeting miR-4443 (Hou et al. 2019). Circ_0020710 is a circRNA that regulates the miR-370-3p/CXCL12 axis in melanoma, mediating cancer progression and immune evasion (Wei et al. 2020). Similarly, Chen et al. (2018) found that hsa_circ_100395 acts as a molecular sponge to regulate the proliferation, migration, and invasion of lung cancer cells through the miR-1228/TCF21 pathway. Currently, the role of the circRNA–microRNA–mRNA axis in CRC remains largely unknown, warranting further research.

In this study, we conducted microarray analysis of circRNAs expression profiles in CRC tissues and successfully constructed a hsa_circ_0074854-mediated CRC miRNA–mRNA regulatory network, which was experimentally validated. Moreover, we combined clinical data and database analysis to verify the results and also explored the relationship between immune infiltration and pathogenic genes. These findings may provide new insights into the molecular mechanisms of colorectal cancer and potential therapeutic targets.

## Materials and methods

### CircRNA microarray and differential expression analysis

CircRNA microarray data from CRC and adjacent normal tissues were collected from the GEO database. The following search terms were used to retrieve circRNA expression profiles for human CRC and adjacent normal tissues: “colorectal cancer” (All Fields) and [“circRNA” (All Fields) or “circular RNA” (All Fields)]. Microarrays with sample counts less than 5 were excluded from the analysis, and datasets from CRC cell lines were also excluded. Finally, we selected GSE197991 and GSE147597, which were based on the same platform (GPL19978). Zero-mean normalization was performed before conducting the differential expression analysis. The R Limma package was used to analyze the differential expression of circRNAs, with |log2(fold change [FC])|≥ 1 and *p < *0.05 as the criteria for identifying DEcircRNAs. The volcano plots and heatmaps illustrating the expression of differentially expressed genes in the samples were generated using the Pheatmap package. Venn analysis was performed to select overlapping DEcircRNAs between the two microarrays.

### CRC tissue specimens

Collection of tissue pairs (including CRC tissues and adjacent normal tissues) was performed from 72 patients at the Second People's Hospital of Lianyungang City. Patient inclusion criteria were as follows: adult patients diagnosed with CRC and no preoperative radiotherapy or chemotherapy. The adjacent normal tissues were collected from the colon tissues located more than 5.0 cm away from the cancer tissues. After retrieval, the specimens were sent to the central laboratory of our institution and stored at − 80 °C in a freezer to prevent circRNA degradation. To ensure minimal circRNA degradation, the time between tissue excision and preservation was strictly controlled within 2 h after tissue removal. This study was approved by the Institutional Review Board (Approval No. 2023k009).

### Cell culture

Human normal colon mucosal cell line (NCM460) and human CRC cell lines (HCT116, HT29, LS174T, and RKO) were purchased from the Shanghai Institute of Biochemistry and Cell Biology (Shanghai, China). These cells were cultured in Roswell Park Memorial Institute-1640 (RPMI-1640) medium (Invitrogen, Carlsbad, CA, USA) supplemented with 10% fetal bovine serum (FBS, Invitrogen, Carlsbad, CA, USA), 100 U/mL penicillin, and 100 μg/mL streptomycin (Sigma, St. Louis, MO, USA), and incubated at 37 °C with 5% CO_2_ in a humidified incubator.

### Cell transfection

The small interfering RNAs (siRNAs) targeting circ_0074854 (si-circ_0074854#1 and si-circ_0074854#2) and siRNA negative control (si-NC) were synthesized by Guangzhou RiboBio Co., Ltd. When HT29 and RKO cells reached 60–70% confluency, the aforementioned siRNAs were transfected into the cells using Lipofectamine 3000 (Invitrogen, USA). After 6 h of transfection, the complete culture medium was replaced, and the cells were further cultured for 48 h before collection.

### RT-qPCR

Total RNA was extracted using TRIzol, and then converted to cDNA using the PrimeScript RT kit and gDNA rubber kit from Takara, Japan. qPCR analysis was performed using the FastStart Universal SYBR Green Master (ROX) from Roche, Germany. The primer sequence is as follows: hsa_circ_0074854, Forward 5ʹ-AAGGGAACCTTTCACTGGTCTG-3ʹ, Reverse 5ʹ-AGAGGCAGCATCTGGCTGAT-3ʹ. hsa-miR-2110, Forward 5ʹ-TGCGGTTGGGGAAACGGCCGCTG-3ʹ, Reverse 5ʹ-CCAGTGCAGGGTCCGAGGT-3ʹ. ENO2, Forward 5ʹ-CGTTACTTAGGCAAAGGTGTCC-3ʹ, Reverse 5ʹ-CTCCAGCATCAGGTTGTCCAGT-3ʹ. IGF2BP3, Forward 5ʹ-TATATCGGAAACCTCAGCGAGA-3ʹ, Reverse 5ʹ-GGACCGAGTGCTCAACTTCT-3ʹ. GAPDH, Forward 5ʹ-AGAAGGCTGGGGCTCATTTG-3ʹ, Reverse 5ʹ-AGGGGCCATCCACAGTCTTC-3ʹ. U6, Forward 5ʹ-CTCGCTTCGGCAGCACAT-3ʹ, Reverse 5ʹ-TTTGCGCTGTCATCCTTGCG-3ʹ.

### CCK-8 experiments detect cell proliferation

48 h after transfection, the transfected cells were seeded into a 96-well plate at a density of 2000 cells per well for the CCK-8 assay. Subsequently, 10 μL of CCK-8 solution (Zomanbio, China) was added to each well, and the plate was kept at 37 °C for 2 h. The absorbance value (OD450) at 450 nm for each well was measured using a multi-functional microplate reader.

### Scratch experiments detect cell migration

When the cell confluency in the 6-well plates reached over 90% after transfection, the original culture medium was discarded under a sterile workbench. A straight line was gently scratched on the 6-well plates using a scratch tool. The cells were washed with PBS buffer until there were no detached cells and then 2 mL of serum-free DMEM medium was added. The cells were observed and photographed under an inverted microscope (0 h time point), and then placed back into the incubator. After 24 h of continued incubation, the cells were observed under an inverted microscope again, and photographs were taken to record the healing of the scratch (24-h time point). The scratch area was analyzed, and the scratch closure rate was calculated.

### Transwell experiment detect cell invasion

For the transwell migration assay, cells were suspended in 200 µL of cell suspension and placed in the upper chambers of a 24-well transwell plate. The lower chambers were filled with DMEM medium containing 10% FBS. After incubating at 37 °C for 24 h, the cells in the upper chambers were gently wiped with a cotton swab. The membrane on the other side of the chamber was fixed with methanol for 15 min and then air dried. The cells were stained with 0.1% crystal violet, and cell counting was performed by capturing photographs.

### Flow cytometry

In the cell cycle experiment, logarithmically growing seed cells were subjected to analysis in a 6-well plate. Cells were cultured in serum-free medium for 24 h until reaching an appropriate cell density. After cell collection, cells were fixed overnight at 4 °C with 70% pre-chilled ethanol. After centrifugation, 400 μL of RNAase solution and PI staining solution were added. The cells were then stained in the dark at room temperature for 30 min.

In the cell apoptosis experiment, trypsin was used to digest the cells into a centrifuge tube. The cells were then washed twice with pre-chilled sterile PBS and adjusted to a concentration of 1 × 10^5^ cells/mL. 200 μL of the cell suspension was taken, and 10 μL of Annexin V-FITC was added, followed by the addition of 10 μL of PI solution to the cell suspension. Subsequently, the cell suspension was incubated in the dark at room temperature for 10 min. Finally, 500 μL of PBS was added. Flow cytometry was used for all analyses and detections.

### CircRNA–miRNA–mRNA network prediction and analysis

To understand the structural model of hsa_circ_0074854, we utilized the Cancer-Specific CircRNAs Database (CSCD). CircBANK and CSCD were employed to predict the sponge miRNAs of hsa_circ_0074854. miRDB and TargetScan were used to predict candidate targets of the selected miRNAs. Hub genes were identified using the Cytoscape plugin "cytoHubba". Overall survival assessment of hub gene expression was conducted through Gene Expression Profiling Interactive Analysis (GEPIA). A significance threshold of *p < *0.05 was used.

### GO and KEGG feature enrichment analysis

The DAVID database is a gene online annotation tool website. The DEmRNA list from the circRNA–miRNA–mRNA network was imported into the DAVID database to obtain the GO and KEGG enrichment analysis results for these genes. GO enrichment includes three categories: molecular function, biological process, and cellular component. Enrichment with a *p* value < 0.05 is considered statistically significant. KEGG consists of four databases: systematic information, genomic information, chemical information, and health information.

### Immune cell infiltration analysis

We performed correlation analysis between the expression of ENO2 and IGF2BP3 in CRC and the levels of immune cell infiltration using the Tumor IMmune Estimation Resource 2.0 (TIMER2.0) database and the Tumor and IMmune System Interaction database (TISIDB). The correlation was assessed by calculating Spearman coefficients based on the gene expression profiles in CRC.

### Statistical analysis

All statistical analyses were performed using IBM SPSS version 23.0. It is important to note that bioinformatics analyses predominantly employed bioinformatics tools and R version 4.0.5. A significance level of *p < *0.05 was considered statistically significant. Graphs were generated using GraphPad Prism 8 (GraphPad Software, La Jolla, CA, USA).

## Results

### Identification of DEcircRNAs in healthy controls and CRC patients.

**Fig. 1:**
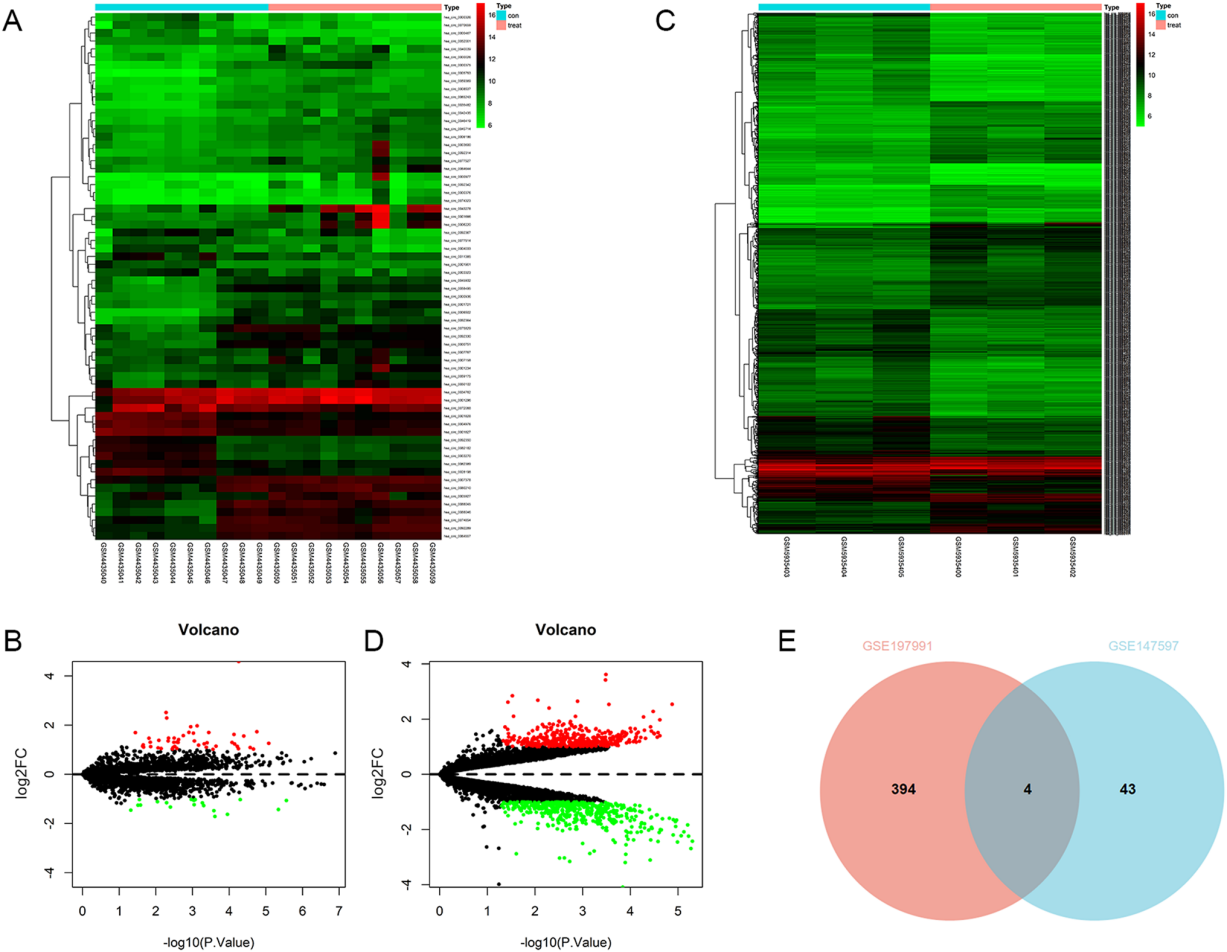
Identification of potential circRNAs in colorectal cancer. **A** The heatmap of differentially expressed circRNAs in colorectal cancer from GSE147597 dataset. **B** The volcano plot of differentially expressed circRNAs in colorectal cancer from GSE147597 dataset. **C** The heatmap of differentially expressed circRNAs in colorectal cancer from GSE197991 dataset. **D** The volcano plot of differentially expressed circRNAs in colorectal cancer from GSE197991 dataset. **E** Venn program showed overlapped upregulated circRNAs in colorectal cancer from GSE147597 and GSE197991 dataset

Comparing with normal samples from healthy controls, GSE147597 identified a total of 66 DEcircRNAs in CRC patients, including 47 upregulated circRNAs and 19 downregulated circRNAs; GSE197991 identified a total of 872 differentially expressed circRNAs in CRC patients, including 398 upregulated circRNAs and 474 downregulated circRNAs (Fig. 1A–D). Four DEcircRNAs were found to be common in the upregulated group from both datasets, namely has_circ_0001234, has_circ_0007158, has_circ_0074854, and has_circ_0058495 (Fig. 1E). Among them, hsa_circ_0074854 was selected for further investigation.

### Has_circ_0074854 are upregulated in CRC cell lines and tissues

**Fig. 2:**
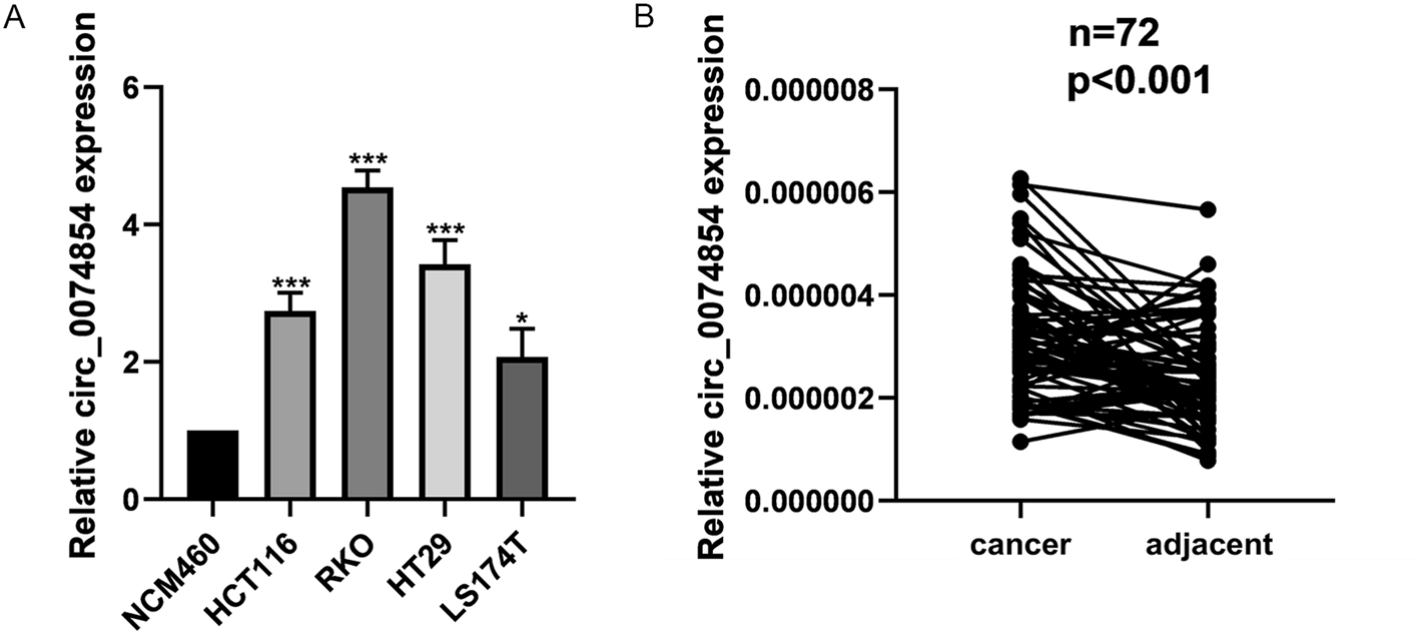
Has_circ_0074854 are upregulated in CRC cell lines and tissues. **A** Relative expression of has_circ_0074854 in a normal colorectal epithelium cell line (NCM460) and CRC cell lines (HCT116, RKO, HT29, and LS174T). **B** RT-qPCR analysis showed that has_circ_0074854 expression was significantly upregulated in 72 CRC tissues compared with paired adjacent normal tissues (*p < 0.05, ***p < 0.001)

The expression of hsa_circ_0074854 was examined in HT29, HCT116, RKO, LS174T, and NCM460 cell lines using RT-qPCR. The results showed that hsa_circ_0074854 was upregulated in all four colorectal cancer cell lines (Fig. 2A). Among them, HT29 and RKO cell lines exhibited a more significant upregulation of hsa_circ_0074854 compared to other cell lines. Therefore, HT29 and RKO cell lines were chosen for further experiments. RT-qPCR was also performed to detect the expression of hsa_circ_0074854 in 72 CRC tissues and adjacent normal tissues. Hsa_circ_0074854 was found to be upregulated in CRC samples compared to cancer adjacent tissues (Fig. 2B).

### Correlation between hsa_circ_0074854 and clinicopathological factors in CRC patients

To explore the relationship between hsa_circ_0074854 and clinicopathological factors in CRC patients, we collected and analyzed data on gender, age, tumor differentiation, tumor size, invasiveness, lymph node metastasis, distant metastasis, and other clinical characteristics of CRC patients. The results indicated that the expression level of hsa_circ_0074854 in colorectal cancer tissue was correlated with tumor differentiation (*p < *0.001), invasiveness (*p* = 0.022), lymph node metastasis (*p < *0.001), and distant metastasis (*p < *0.02), providing direction for subsequent cell experiments (Table 1).

**Table 1:** The association between Hsa_circ_0074854 and clinicopathological factors in CRC patients

Characteristics	Total	Hsa_circ_0074854 expression		*p* value
		Low expression (*n* = 38)	High expression (*n* = 38)	
Gender				0.925
Male	29	16	13	
Female	43	22	21	
Age (years)				0.063
< 60	19	14	5	
≥ 60	53	24	29	
TNM stage				< 0.001
I + II	37	37	0	
III + IV	35	1	34	
T stage				0.022
T1 + T2	28	20	8	
T3 + T4	44	18	26	
N stage				< 0.001
N0	42	37	5	
N1 + N2	30	1	29	
M stage				0.02
M0	67	38	29	
M1	5	0	5	
Diameter (cm)				0.702
< 4	26	15	11	
≥ 4	46	23	23	

### The effect of hsa_circ_0074854 knockdown on the proliferation ability of colorectal cancer cells

**Fig. 3:**
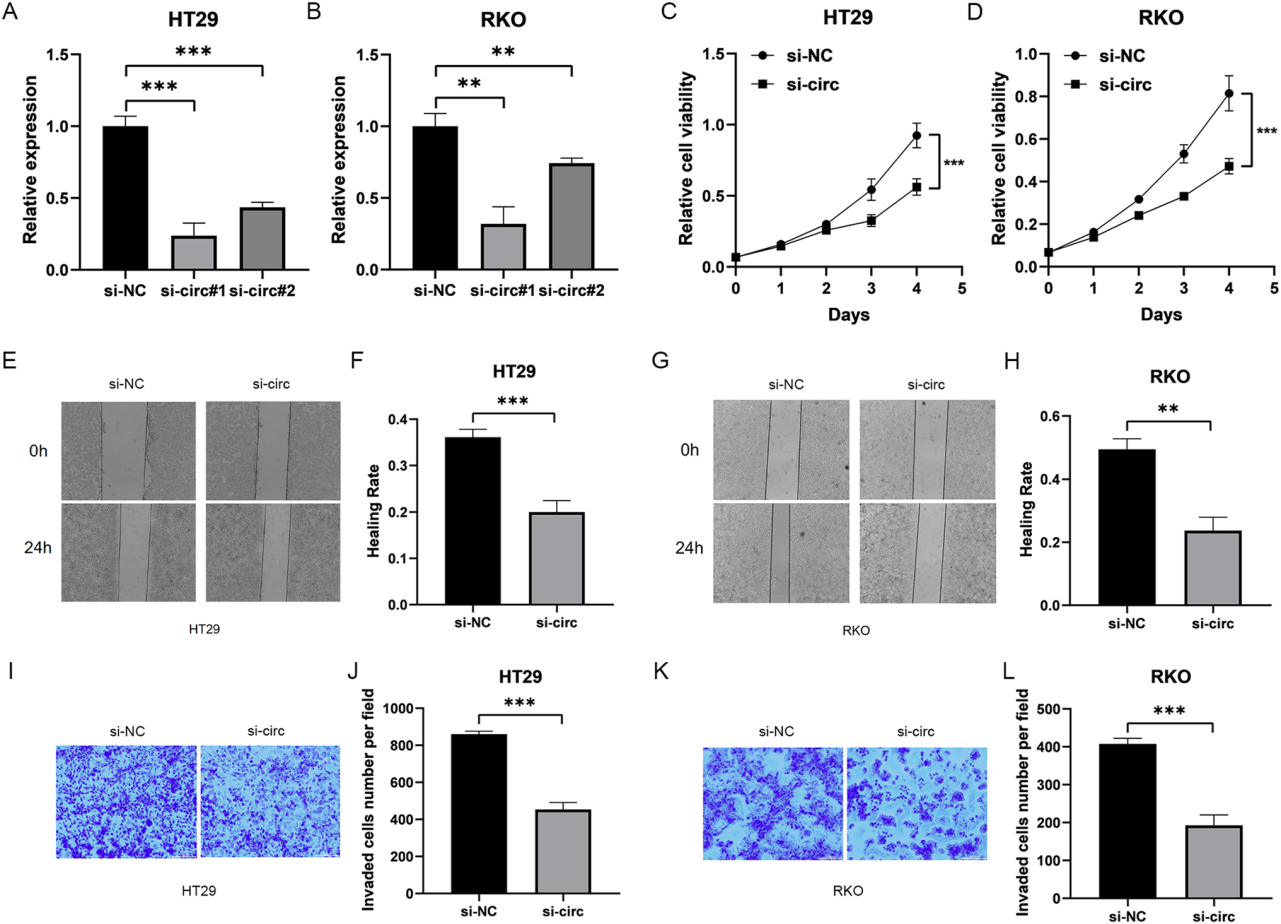
Knockdown of hsa_circRNA_0074854 inhibits the proliferation, migration and invasion of CRC cells. **A** and **B** The expression level of hsa_circ_0074854 after transfecting small interfering RNAs. **C** and **D** HT29 cells and RKO cells were transfected with hsa_circ_0074854-siRNA. CCK-8 was utilized to measure proliferation rate. The effect of hsa_circ_0074854 knockdown on the migration of colorectal cancer cells. **E**–**H** RKO cells and HT29 cells were transfected with hsa_circ_0074854-siRNA. Cell migration was measured with the wound healing assay. **I**–**L** HT29 cells and RKO cells were transfected with hsa_circ_0074854-siRNA.Transwell invasion assays were performed (*p < 0.05 and **p < 0.01)

As hsa_circ_0074854 was upregulated in CRC cells and tissues, suggesting its potential role in promoting CRC progression, we designed siRNAs to silence hsa_circ_0074854 and transfected them into HT29 and RKO cells. After transfection of siRNA#1 and siRNA#2 in HT29 and RKO cell lines, the expression of hsa_circ_0074854 decreased by approximately 70% and 40%, respectively (Fig. 3A, B). Subsequently, we chose siRNA#1, which showed better knockdown efficiency, for further experiments. Using the CCK-8 assay, we assessed the effect of hsa_circ_0074854 on the proliferation of CRC cells and found that its knockdown significantly inhibited the proliferation of colorectal cancer cells (Fig. 3C, D).

### The effect of hsa_circ_0074854 knockdown on the migration ability of colorectal cancer cells

After cell transfection, the effect of hsa_circ_0074854 on cell migration was examined using a scratch assay. The results of the scratch assay showed that the migration rates (%) of the experimental group were approximately 0.35 (HT29) and 0.49 (RKO) after si-circ transfection, while the migration rates (%) of the control group were approximately 0.19 (HT29) and 0.21 (RKO). These findings indicate that the downregulation of hsa_circ_0074854 significantly inhibits the migration of colorectal cancer cells (Fig. 3E–H).

### The effect of hsa_circ_0074854 knockdown on the invasion ability of colorectal cancer cells

To evaluate the invasive ability, we performed transwell cell invasion assays on HT29 and RKO cells transfected with si-NC and si-circ. The results showed that the control group had approximately 400 and 840 cells per field for HT29 and RKO, respectively, while the experimental group had approximately 190 and 430 cells per field. These results indicated that the knockdown of hsa_circ_0074854 significantly suppressed the invasive ability of colorectal cancer cells (Fig. 3I–L).

### The effect of hsa_circ_0074854 knockdown on cell cycle and apoptosis of colorectal cancer cells

**Fig. 4:**
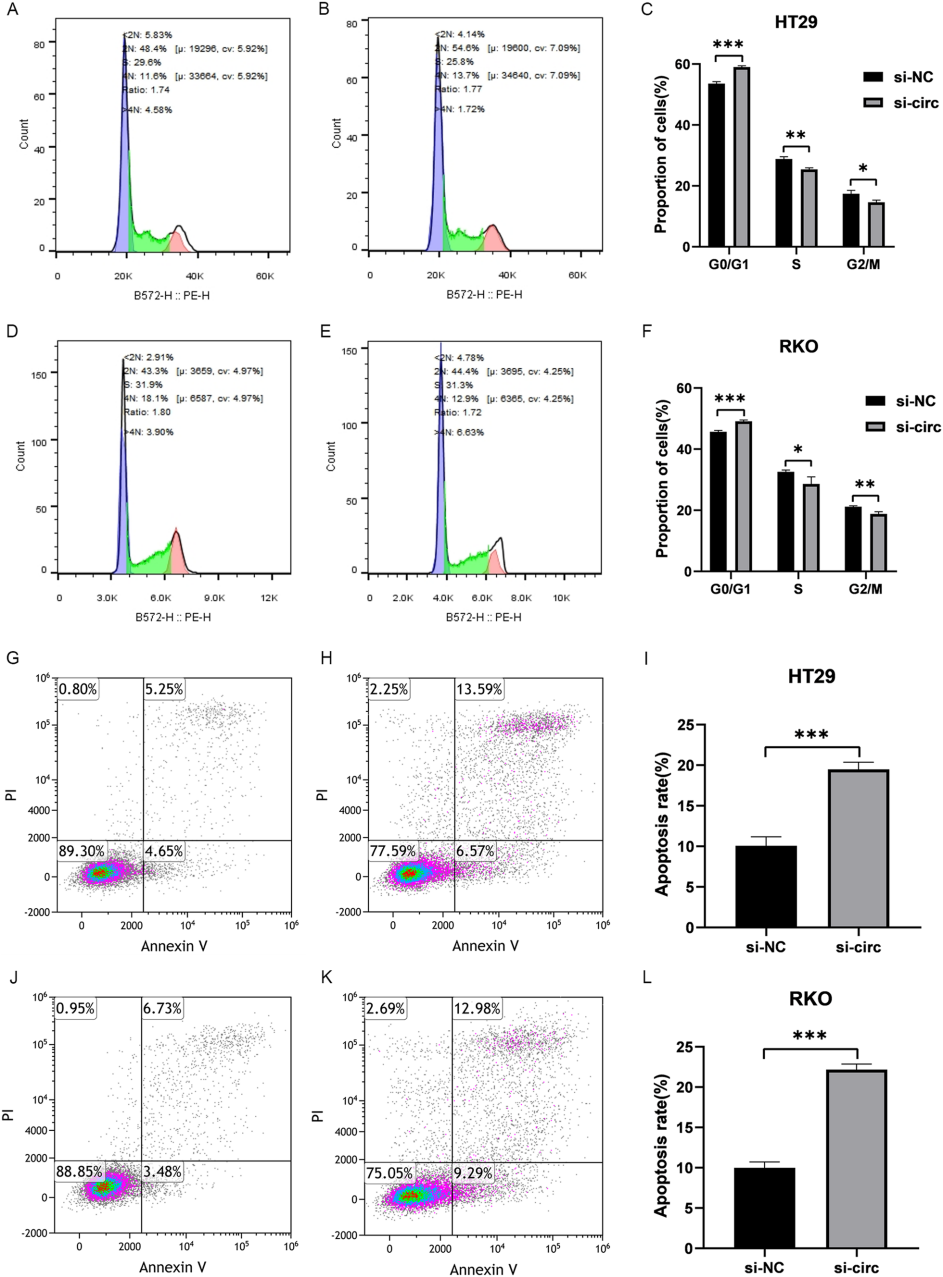
The Effect of hsa_circ_0074854 knockdown on apoptosis and cell cycle of colorectal cancer cells. **A**–**F** Flow cytometry was carried out to check the cycle in HT29 and RKO. **G**–**L** Flow cytometry was carried out to check the apoptosis rate in HT29 and RKO (*p < 0.05, **p < 0.01, ***p < 0.001)

We performed flow cytometry to analyze the cell cycle distribution of HT29 and RKO cells after transfection with hsa_circ_0074854 siRNA. The results showed that the proportion of G0/G1 phase cells in the si-NC group was 46.21% and 54.23% for HT29 and RKO, respectively; while in the si-circ group, it was 49.18% and 58.74%. This upregulation of cells in the G0/G1 phase indicated cell cycle delay and cell cycle arrest (Fig. 4A–F). Additionally, compared to the si-NC group, the knockdown of hsa_circ_0074854 significantly improved cell apoptosis. Further analysis using flow cytometry demonstrated that the level of apoptosis in HT29 and RKO cells treated with hsa_circ_0074854 knockdown was significantly lower (5.26% and 4.01%, respectively) than that in the si-NC control group, with statistically significant differences (Fig. 4G–L).

### Bioinformatics analysis and prediction of hub genes

**Fig. 5:**
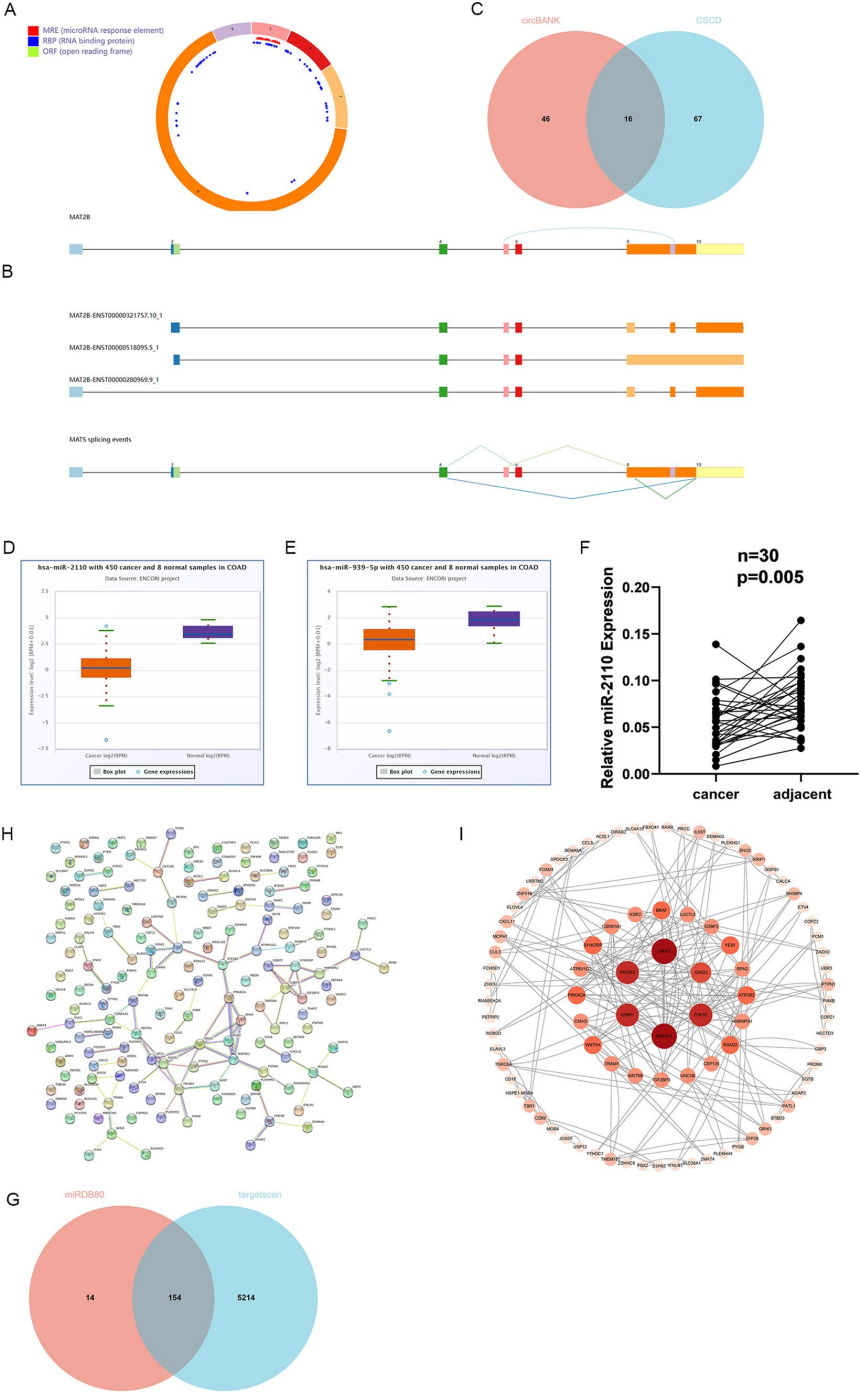
Identification of potential miRNAs that bind to circRNAs in colorectal cancer and prediction of hub genes. **A** and **B** Structural patterns of the hsa_circ_0074854 in CSCD database. **C** Venn program showed overlapped downregulated miRNAs in colorectal cancer from circBANK and CSCD databases. **D** The expression level of hsa-miR-2110 from ENCORI. **E** The expression level of hsa-miR-939-5p from ENCORI. **F** Relative hsa-miR-2110 levels were measured in tumor adjacent tissues and CRC tissues. **G** The Venn program showed that cross-analysis of miRDB and TargetScan databases predicted miR-2110 target gene overlap. **H** A protein–protein interaction (PPI) network of 154 mRNAs. **I** 91 Hub genes were identified by the CytoHubba

To investigate the molecular mechanisms promoting colorectal cancer progression, we first obtained information about hsa_circ_0074854 from the CSCD (Fig. 5A, B). We found that hsa_circ_0074854 is an exon-circRNA, suggesting that it may act as a miRNA sponge. Using circBANK and CSCD databases, we predicted potential target miRNAs for hsa_circ_0074854, resulting in 62 and 83 miRNAs, respectively. The intersection of these two sets yielded 16 miRNAs (Fig. 5C). Among them, only miR-2110 and miR-939-5p were downregulated (Fig. 5D, E). Since further analysis of miR-939-5p's target genes, identified through database prediction and screening for central genes, did not meet both overall survival rate and expression relationship, it was not pursued in further studies. We observed higher expression of miR-2110 in cancer tissues compared to tumor adjacent tissues (Fig. 5F).

Using TargetScan and miRDB, we predicted potential target mRNAs for miRNAs. In the miRDB database, higher Target Score indicates higher credibility, typically considered reliable when Target Score is greater than 80. Thus, miR-2110 yielded 5368 and 168 potential target mRNAs, respectively. The intersection of these two sets identified 154 mRNAs. We revealed the protein–protein interaction (PPI) network of the 154 mRNAs using the STRING database (Fig. 5I). The central genes among the 154 mRNAs were identified using the CytoHubba plugin, totaling 91 hub genes for further analysis (Fig. 5G).

### mRNA function analysis

**Fig. 6:**
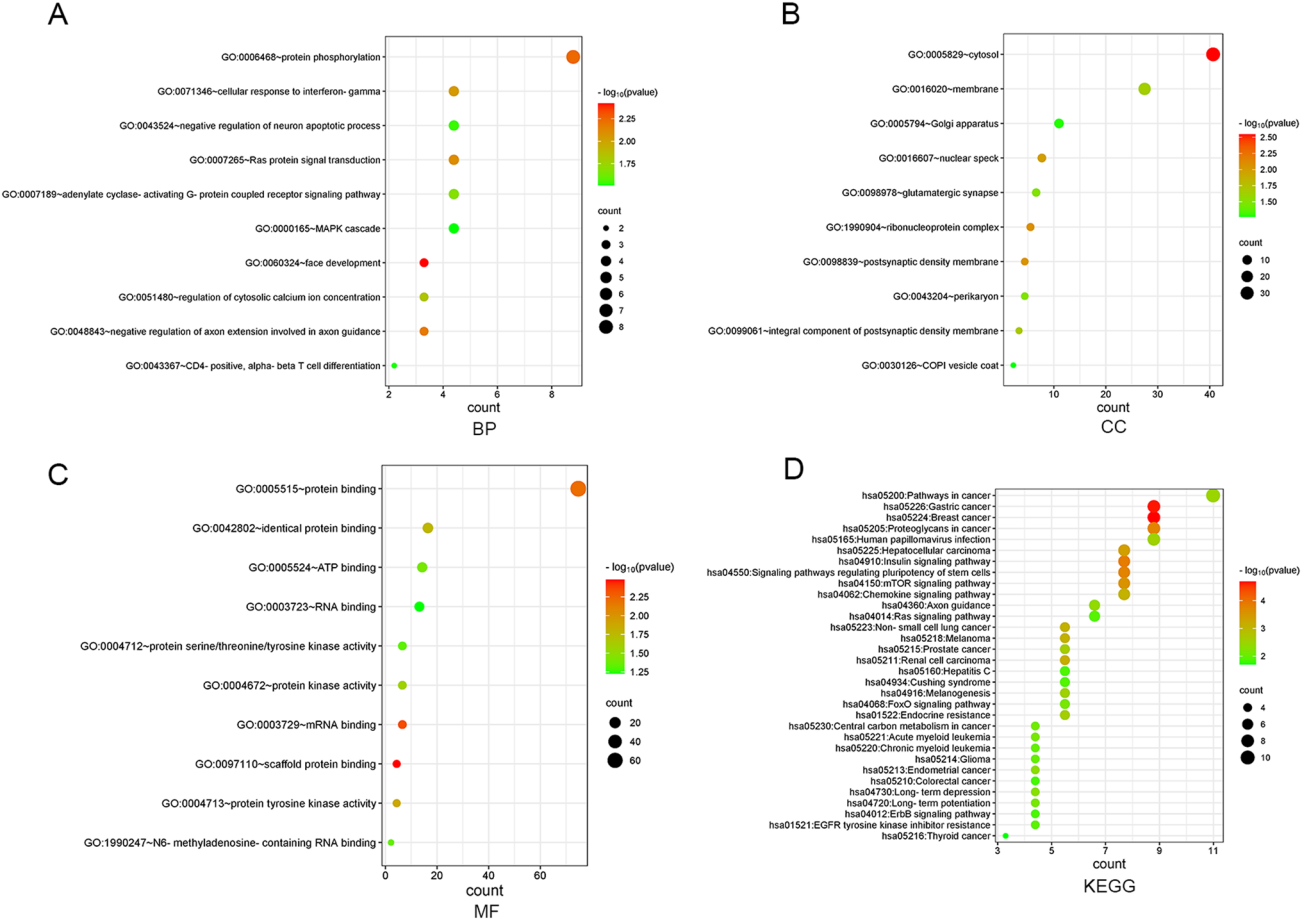
Function analysis. **A**, **B**, and **C** GO function analysis of hub genes. **D** KEGG function analysis of hub genes

To elucidate the potential biological functions of hsa_circ_0074854 in CRC development, we conducted GO and KEGG enrichment analysis on the predicted 91 hub genes. GO bio-process (BP) analysis revealed that the target genes are mainly involved in processes such as protein phosphorylation, ras protein signal transduction, MAPK cascade, CD4-positive, alpha–beta T cell differentiation, and more (Fig. 6A). In cell composition (CC) analysis, the target genes are significantly enriched in the cytosol and membrane (Fig. 6B). Furthermore, molecular functional (MF) analysis indicated that these mRNAs are mainly associated with protein binding, ATP binding, and RNA binding (Fig. 6C). Additionally, KEGG enrichment analysis demonstrated significant correlations between the differentially expressed mRNAs and various cancer-related pathways, including pathway in cancer, gastric cancer, breast cancer, colorectal cancer, human papillomavirus infection, proteoglycans in cancer, signaling pathways regulating pluripotency of stem cells, mTOR signaling pathway, chemokine signaling pathway, non-small-cell lung cancer, and others (Fig. 6D). These findings may provide valuable references for further research.

### Construction of circRNA–miRNA–mRNA network

**Fig. 7:**
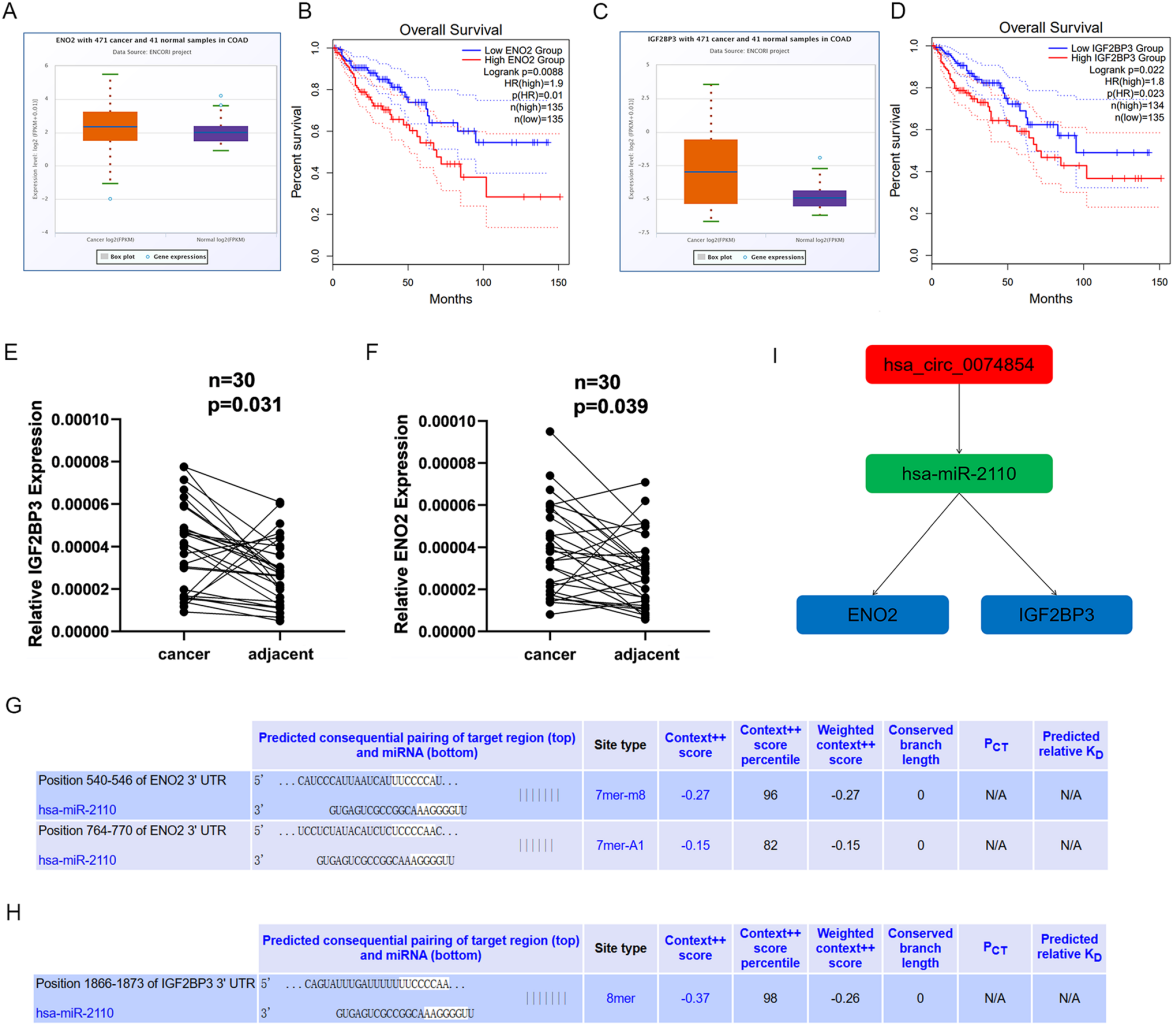
Construction of the circRNA–miRNA–mRNA network of hsa_circ_0074854 in CRC. **A**, **C** The expression level of ENO2 and IGF2BP3 from ENCORI. **B**, **D** Overall survival curves of ENO2 and IGF2BP3 from ENCORI. **E**, **F** Relative ENO2 and IGF2BP3 levels were measured in tumor adjacent tissues and CRC tissues. **G**, **H** The potential binding sites of miR-2110 to ENO2 and IGF2BP3. **I** A circRNAs–miRNAs–mRNAs network

We assessed the impact of the expression of the 91 central genes targeted by miR-2110 on the overall survival of CRC using the GEPIA database. We found that two of the central genes (ENO2 and IGF2BP3) were upregulated in CRC and negatively correlated with overall survival (Fig. 7A–D). The expression of ENO2 and IGF2BP3 in cancer tissues was higher than that in adjacent normal tissues (Fig. 7E, F). Moreover, we identified potential binding sites of miR-2110 with ENO2 and IGF2BP3 using online databases (Fig. 7J, H), providing references for subsequent experiments. Based on the central genes with prognostic potential, we constructed a possible ceRNA visualization network comprising one circular RNA (hsa_circ_0074854), one miRNA (miR-2110), and two mRNAs (ENO2 and IGF2BP3) (Fig. 7I).

### Expression of miRNA and mRNA after knockout of has_circ_0074854 in CRC cells

**Fig. 8:**
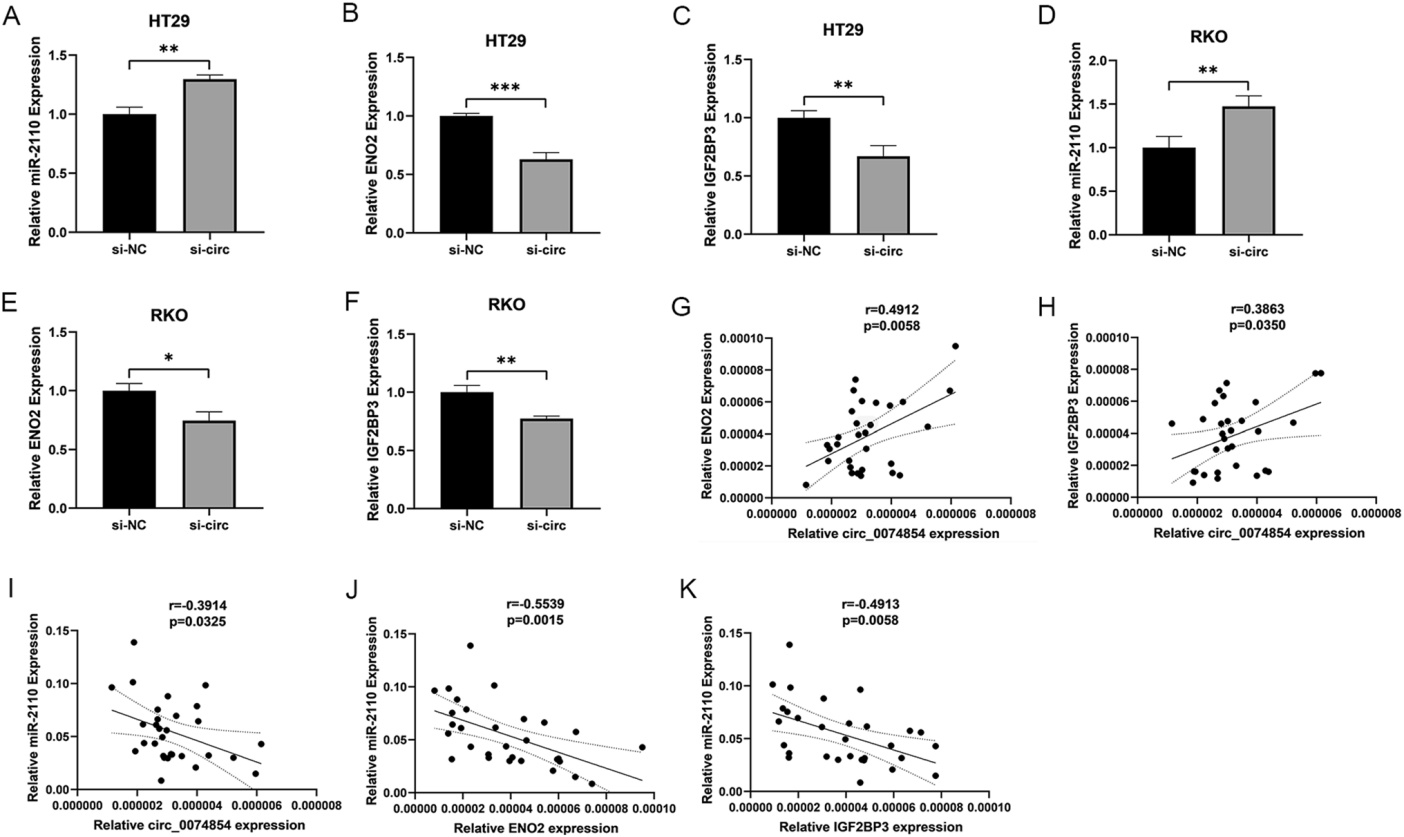
The expression of miRNAs and mRNA in knockdown of hsa_circ_0074854 CRC cells and correlation between circRNA, miRNA, and mRNA. **A**–**C** The relative expressions of miR-2110 in knockdown of hsa_circ_0074854 CRC cells. **D**–**F** The relative expressions of ENO2 in knockdown of hsa_circ_0074854 CRC cells. **G** The relative expressions of IGF2BP3 in knockdown of hsa_circ_0074854 CRC cells. **H** The expression of circ_0074854 was positively correlated with ENO2 and IGF2BP3 expression. **I** The expression of circ_0074854 was negatively correlated with miR-2110 expression. **J** and **K** The expression of miR-2110 was negatively correlated with ENO2 and IGF2BP3 expression (*p < 0.05, **p < 0.01, ***p < 0.001)

To further validate the potential ceRNA network of hsa_circ_0074854, we examined the expression levels of miR-2110, ENO2, and IGF2BP3 in siRNA-hsa_circ_0074854-transfected HT29 and RKO cells. In siRNA-hsa_circ_0074854-transfected HT29 cells, the expression of miR-2110 was significantly upregulated; while, ENO2 and IGF2BP3 were significantly downregulated (Fig. 8A–C). Similarly, in siRNA-hsa_circ_0074854-transfected RKO cells, miR-2110 expression was significantly upregulated; while, ENO2 and IGF2BP3 expression were significantly downregulated (Fig. 8D–F).

### Correlation between circRNA, miRNA, and mRNA

**Fig. 9:**
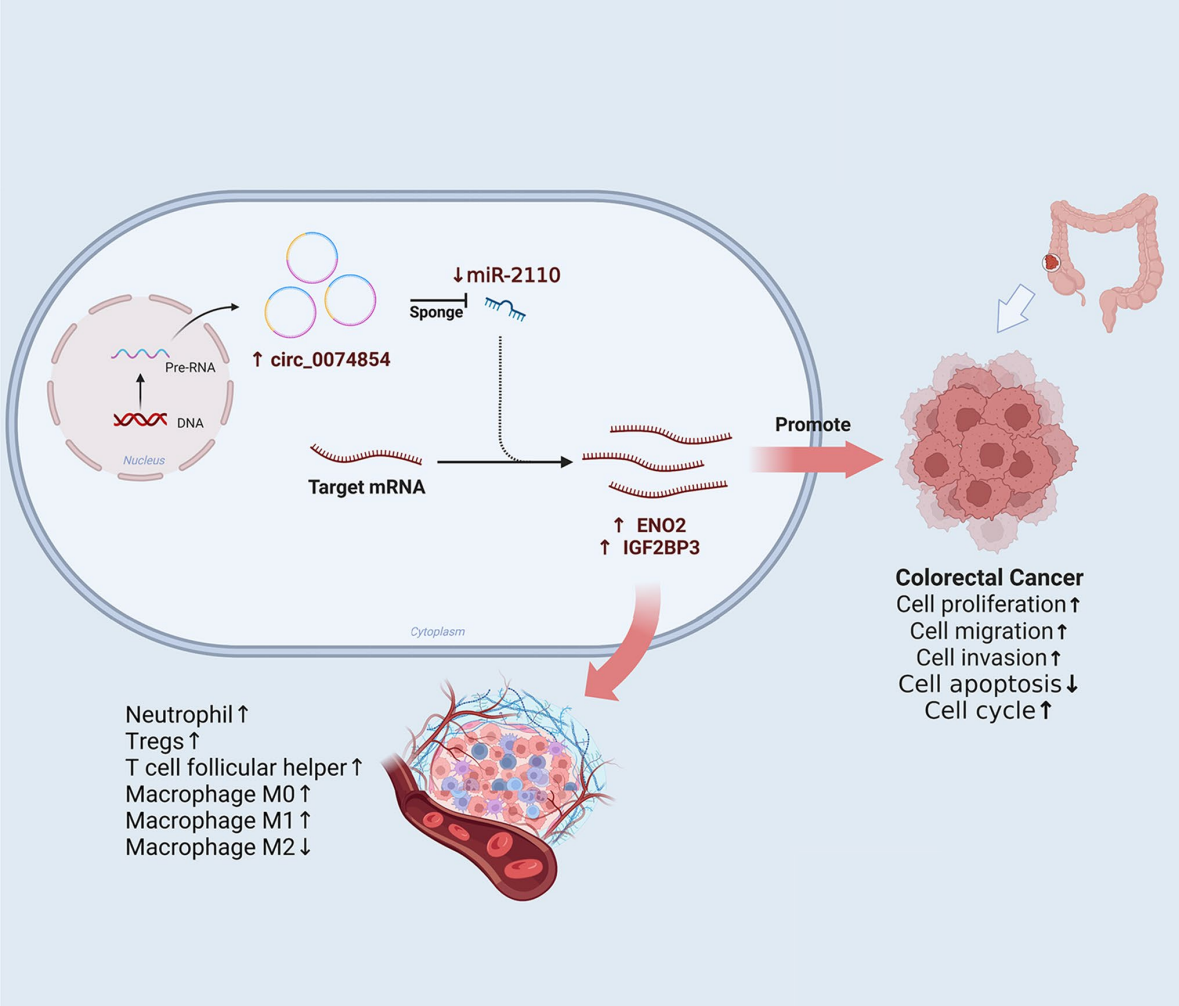
Hsa_circ_0074854 exerts its effects by suppressing miR-2110, leading to the upregulation of ENO2/IGF2BP3, which in turn promotes colorectal cancer (CRC) cell proliferation, migration, invasion, and cell cycle progression, while inhibiting CRC cell apoptosis, and is accompanied by immune cell infiltration

We examined the expression of has_circ_0074854, miR-2110, ENO2, and IGF2BP3 in 30 colorectal cancer tissues and investigated their interrelationships. Significant correlations were observed between them in both CRC tissues and adjacent normal tissues. The expression of has_circ_0074854 positively correlated with ENO2 and IGF2BP3 (Fig. 8G, H), while it negatively correlated with miR-2110 (Fig. 8I). Conversely, miR-2110 expression negatively correlated with ENO2 and IGF2BP3 (Fig. 8J, K). These results are consistent with the data from the aforementioned databases, further confirming the reliability of constructing the circRNA–miRNA–mRNA network. Taken together, hsa_circ_0074854 exerts its effects by suppressing miR-2110, leading to the upregulation of ENO2/IGF2BP3, which in turn promotes colorectal cancer (CRC) cell proliferation, migration, invasion, and cell cycle progression, while inhibiting CRC cell apoptosis, and is accompanied by immune cell infiltration (Fig. 9).

### Correlations between gene expression and immune cell infiltration in CRC

**Fig. 10:**
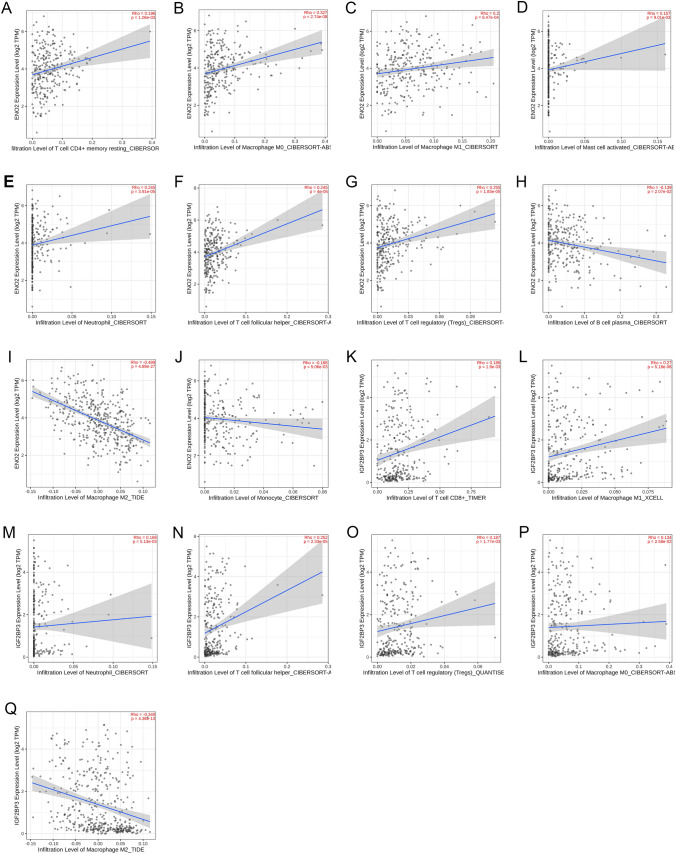
Correlations between gene expression and immune cell infiltration in CRC. **A** ENO2–T cell CD4 + memory resting. **B** ENO2–macrophage M0. **C** ENO2–macrophage M1. **D** ENO2–mast cell activated. **E** ENO2–neutrophil. **F** ENO2–T cell follicular helper. **G** ENO2–Tregs. **H** ENO2–B cell plasma. **I** ENO2–macrophage M2. **J** ENO2–monocyte. **K** IGF2BP3–T cell CD8 + . **L** IGF2BP3–macrophage M1. **M** IGF2BP3–neutrophil. **N** IGF2BP3–T cell follicular helper. **O** IGF2BP3–Tregs. **P** IGF2BP3–macrophage M0. **Q** IGF2BP3–macrophage M2

To determine the impact of differentially expressed immune-related genes on the tumor microenvironment (TME), we conducted immune cell infiltration analysis. We found that ENO2 expression positively correlated with the infiltration of T cell CD4 + memory resting, neutrophil, T cell follicular helper, mast cell activated, Tregs, macrophage M0, and macrophage M1 (Fig. 10A–G); while, it negatively correlated with B cell plasma, monocyte, and macrophage M2 (Fig. 10H–J). Similarly, IGF2BP3 expression positively correlated with the infiltration of T cell CD8 + , neutrophil, T cell follicular helper, Tregs, macrophage M0, and macrophage M1 (Fig. 10K–P); while, it negatively correlated with macrophage M2 (Fig. 10Q). Notably, neutrophil, Tregs, T cell follicular helper, macrophage M0, macrophage M1, and macrophage M2 showed common correlations with the expression of both ENO2 and IGF2BP3. All these findings are statistically significant.

## Discussion

CRC is currently one of the most well-known malignant tumors worldwide, posing significant challenges in diagnosis and treatment (Hu et al. 2020). Studies have revealed the presence of thousands of circRNAs in human peripheral blood through RNA sequencing (Memczak et al. 2015), indicating their potential value in disease diagnosis. So far, numerous circRNAs have been identified and confirmed to play important roles in tumorigenesis, including lung cancer (Hua et al. 2022), bladder cancer (Lv et al. 2023), glioma(Jiang et al. 2022), gastric cancer(Zang et al. 2022), and other malignancies, where abnormal circRNA expression has been observed. In recent years, many studies have been dedicated to finding biomarkers for colorectal cancer and improving prognosis and treatment strategies. For instance, Jian et al. (2020) demonstrated that CircRNA_001680 enhances CRC cell proliferation and migration, while circRHOBTB3 inhibits CRC cell growth and metastasis (Chen et al. 2021a, b). In this study, through GEO databases and microarray analysis, we found abnormal expression of has_circ_0074854 in CRC. Further investigations revealed elevated expression of has_circ_0074854 in CRC cells and tissues. Silencing has_circ_0074854 significantly inhibited cell proliferation, migration, invasion, and promoted apoptosis in CRC cells, suggesting its role as an oncogenic factor in CRC. Wang et al.'s study unveiled that the downregulation of hsa_circ_0074854 suppresses the migration and invasion in hepatocellular carcinoma via interacting with HuR and via suppressing exosomes-mediated macrophage M2 polarization (Wang et al. 2021a, b, c). Previous research has indicated that circRNAs can be transported from cancer cells to recipient cells within the tumor microenvironment (TME) via exosomes (Huang et al. 2020; Bian et al. 2019), a process crucial for tumor progression (Pascut et al. 2020). Wang et al.'s research underscores the significant role played by has_circ_0074854 in liver cancer. However, the function of has_circ_0074854 in CRC has not been reported previously.

While some studies have reported regulatory networks involving circRNAs in CRC (Liu et al. 2022; Ding et al. 2020; Xing et al. 2020; Sakshi et al. 2021), the understanding of the circRNA-related competing endogenous RNA (ceRNA) network in CRC remains limited and requires further exploration. In this study, through a series of database analyses, we have identified a potential circRNA–miRNA–mRNA ceRNA regulatory network involved in the pathogenesis of CRC. After performing differential expression analysis on the database data, we identified four circRNAs that met the criteria, and we selected has_circ_0074854 for further investigation. Multiple studies have demonstrated that circRNAs can act as miRNA sponges to positively regulate downstream gene expression (Sang et al. 2019; Wang et al. 2021a, b, c; Liu et al. 2019; Ji et al. 2022; Hu et al. 2022). Therefore, we predicted the potential miRNAs targeted by circRNA. Subsequently, we further explored the detailed downstream molecular mechanisms of circRNA–miRNA in CRC. It is well known that miRNAs exert their functions through negative regulation of gene expression (Ding et al. 2020). We then predicted potential miRNA-targeted mRNAs through TargetScan and miRDB, and after intersecting the results, we performed a series of analyses, including miRNA and mRNA expression analysis, prognosis analysis, and finally identified the two most significant targets of miR-2110 (ENO2 and IGF2BP3); while, miR-939-5p had no downstream targets that met the requirements. We measured the expression levels of miR-2110, ENO2, and IGF2BP3 in tissues, and compared to tumor adjacent tissues, the expression of miR-2110 was decreased; while, ENO2 and IGF2BP3 were upregulated in cancer tissues. According to the ceRNA mechanism, there should be a positive expression relationship between circRNA and target genes, a negative correlation between circRNA and miRNA, and a negative correlation between miRNA and target genes. Our results were consistent with these relationships, as the expression of hsa_circ_0074854 positively correlated with ENO2 and IGF2BP3, while the expression of circ_0074854 negatively correlated with mir-2110, and the expression of mir-2110 negatively correlated with ENO2 and IGF2BP3. Interestingly, Yang et al. (2022) found that miR-140-3p can inhibit the translation of FOXQ1 without the need for FOXQ1 mRNA degradation, which differs from the results of many other studies (Chen et al. 2021a, b; Huang et al. 2021). The relative roles of translation inhibition and mRNA degradation, and whether these pathways are sequential or parallel, remain controversial. Existing research has confirmed that translation inhibition can occur without mRNA degradation (Djuranovic et al. 2012; Bazzini et al. 2012; Béthune et al. 2012). The mismatch between mRNA and protein levels suggests that hsa_circ_0088036 may indirectly regulate FOXQ1 expression. Therefore, Yang et al.'s study also provides new insights into miRNA-mediated repression.

After constructing the network, we further analyzed the immune infiltration related to ENO2 and IGF2BP3 in CRC, observing a connection between gene expression and immune cell infiltration. Although previous studies have reported that the behavior and prognosis of tumor patients may be influenced by immune infiltration (Zhu et al. 2023), the interaction mechanism between ENO2, IGF2BP3, and the tumor microenvironment (TME) in CRC remains unclear. Our analysis results indicated that the expression of ENO2 and IGF2BP3 was positively correlated with neutrophil, Tregs, T cell follicular helper, macrophage M0, and macrophage M1; while it was negatively correlated with macrophage M2. Some neutrophil populations, known as tumor-associated neutrophils (TANs), promote cancer cell growth, invasion, and angiogenesis by producing matrix metalloproteinase-9 (MMP9), vascular endothelial growth factor (VEGF), and hepatocyte growth factor (HGF) at primary and metastatic sites (Geh et al. 2022). Macrophages, as immune cells infiltrating the TME with early appearance and abundant numbers, play a crucial role in tumor progression. Macrophages have different subtypes and differentiate under the influence of various cytokines. Based on their activation states, macrophages can be classified into M0, M1, and M2 subtypes, each with distinct immunological functions. M0 is an inactive subtype that does not exhibit inflammatory or tumor-related functions. Depending on the activation pathways, M0 can differentiate into M1- and M2-activated subtypes, each showing different characteristics. M1 macrophages secrete pro-inflammatory cytokines such as IL-12, IL-16, and INF-γ, which activate inflammatory responses and participate in host innate immunity to eliminate tumor cells in the TME. On the other hand, M2 macrophages participate in Th2-type immune responses, suppress T cell proliferation and differentiation, and promote tumor cell proliferation and angiogenesis in the tumor stroma (Mantovani et al. 2005; Hume 2015; Gordon and Martinez 2010; Pollard 2004). Tregs, a heterogeneous population, can stimulate and suppress immune responses based on their phenotype and marker expression. Tregs have been shown to possess T cell suppressive functions in CRC (Bergsland et al. 2022). In pathogen clearance and vaccine-mediated immune processes, TFH is essential for maintaining protective antibody responses. Conversely, abnormal and excessive TFH cell responses mediate and sustain pathogenic antibodies against self-antigens, alloantigens, and allergens, leading to lymphoma and even viral host responses (Yu et al. 2022). Therefore, the elevated expression of ENO2 and IGF2BP3 in CRC may influence tumor immunity and contribute to carcinogenesis, providing implications for future research on immunotherapy. Further experiments can be conducted in future studies to explore relevant mechanisms. This also suggests that the treatment of diseases can not only focus on molecular aspects but can also be combined with immune infiltration directions to achieve better effects.

Therefore, besides the ceRNA mechanism, further research is required to explore the involvement of circRNAs in new domains. The functional elucidation of the established network in CRC requires more efforts in both in vitro and in vivo experiments. New effective methods and bioinformatics tools are needed to predict circRNA target genes. The circRNA–miRNA–mRNA regulatory network must be refined to reveal the pathogenic mechanisms and identify novel diagnostic biomarkers. With advancements in research and technology, the functions of most circRNAs will be unveiled and applied in human cancers. Due to the conservation, stability, specificity, and abundance of circRNAs in blood, saliva, and other body fluids (Xue et al. 2022), they can be detected clinically, suggesting their potential use as non-invasive biomarkers. In this study, hsa_circ_0074854 showed positive correlations with tumor size, lymph node metastasis, and distant metastasis in CRC. Patients with higher hsa_circ_0074854 expression had poorer overall survival (OS). Therefore, hsa_circ_0074854 may serve as an ideal novel diagnostic and prognostic biomarker for CRC. In the future, extensive clinical data on CRC should be utilized to assess the diagnostic and prognostic values of each RNA in the established network, which will contribute to the development of promising diagnostic and prognostic biomarkers for CRC patients.

## Conclusion

In summary, this study has revealed a potential hsa_circ_0074854-mediated circRNA–miRNA–mRNA ceRNA network in CRC through a series of database analyses, including differential expression analysis, cross-analysis, correlation analysis, and partial experimental validation. Furthermore, the study has explored the relationship between mRNA expression levels and immune infiltration, which may impact patients’ treatment response, such as immunotherapy. Although further experimental and clinical validation is required, targeting molecules within this network could potentially serve as diagnostic and therapeutic biomarkers for the disease, offering a novel approach to diagnose and treat CRC patients.

## Data Availability

The datasets used during the current study are available from the corresponding author on reasonable request.
